# Designing Click One-Pot Synthesis and Antidiabetic Studies of 1,2,3-Triazole Derivatives

**DOI:** 10.3390/molecules28073104

**Published:** 2023-03-30

**Authors:** Kainat Shafique, Aftab Farrukh, Tariq Mahmood Ali, Sumera Qasim, Laila Jafri, Hisham S. M. Abd-Rabboh, Murefah mana AL-Anazy, Saima Kalsoom

**Affiliations:** 1 SA-Center for Interdisciplinary Research in Basic Sciences, International Islamic University, Islamabad 44000, Pakistan; 2 Department of Physics, PMAS-Arid Agriculture University, Rawalpindi 44000, Pakistan; 3 Health Services Academy, Islamabad 44000, Pakistan; 4 Department of Pharmacology, College of Pharmacy, Jouf University, Sakaka 72431, Saudi Arabia; 5 Department of Life Sciences, Abasyn University, Islamabad Campus, Islamabad 44000, Pakistan; 6 Chemistry Department, Faculty of Science, King Khalid University, P.O. Box 9004, Abha 61413, Saudi Arabia; 7 Department of Chemistry, College of Sciences, Princess Nourah bint Abdulrahman University (PNU), P.O. Box 84428, Riyadh 11671, Saudi Arabia; 8 Department of Chemistry, PMAS-Arid Agriculture University, Rawalpindi 44000, Pakistan

**Keywords:** triazole derivatives, diabetes mellitus, antidiabetic assay molecular docking

## Abstract

In the present study, a new series of 1,2,3-triazole derivatives was synthesized via a click one-pot reaction. The synthesized compounds were found to be active during molecular docking studies against targeted protein 1T69 by using the Molecular Operating Environment (MOE) software. The designed and synthesized compounds were characterized by using FT-IR, ^1^H-NMR and LC-MS spectra. The synthesized triazole moieties were further screened for their α-amylase and α-glucosidase inhibitory activities. The preliminary activity analysis revealed that all the compounds showed good inhibition activity, ranging from moderate to high depending upon their structures and concentrations and compared to the standard drug acarbose. Both in silico and in vitro analysis indicated that the synthesized triazole molecules are potent for DM type-II. Out of all the compounds, compound K-1 showed the maximum antidiabetic activity with 87.01% and 99.17% inhibition at 800 µg/mL in the α-amylase and α-glucosidase inhibition assays, respectively. Therefore these triazoles may be further used as promising molecules for development of antidiabetic compounds.

## 1. Introduction

Heterocycles containing nitrogen atoms indicate a main pharmacophore system in a wide range of pharmaceutical materials [1]. The good binding interactions between the nitrogen atoms in the heterocycles and the targeted inhibitors result in improved pharmacokinetics and metabolism [2]. Triazoles, nitrogen-containing heterocycles, are considered to be one of the most important nitrogen-containing moieties. The 1,2,3-triazole ring system is an aromatic five-membered heterocycle containing three adjacent nitrogen atoms as one of two isomeric structures with the molecular formula C_2_N_3_H_3_ [3]. The 1,2,3-triazole ring system can form hydrogen bonds, dipole–dipole bonds and Van der Waals forces interactions with various biomolecules, such as enzymes, proteins, nucleic acids and other receptors [4]. Thus, compounds containing the 1,2,3-triazole ring system have been used as agents for different biological activities, such as antimicrobial [5], anticancer [6], antioxidant [7], antiviral and antidiabetic activities [8]. Diabetes is a group of chronic metabolic disorders characterized by hyperglycemia resulting from defects in the secretion and action of the insulin hormone. There are different types of diabetes, but the most common ones are type-I and type-II diabetes. In type-I diabetes, the body’s immune system attacks and destroys the cells in the pancreas that produce insulin, while in type-II diabetes, the body becomes resistant to the effects of insulin or does not produce enough insulin to regulate blood sugar levels properly. Diabetes is associated with a series of complex and chronic disorders characterized by indicative glucose intolerance and ensuing from absolute or relative imbalanced insulin secretion or insulin action [9]. The substrate side chain conformation present in triazoles and other moieties impacts reactivity during glycosylation and glycoside hydrolysis and is restricted by many glycosidases and glycosyltransferases during catalysis [10]. The other heteroatoms present in the structures, such as oxygen, play an important role in inhibition activity [11]. N-glycopeptides, sugar-derived triazoles and their structures are of great importance as amino sugars [12]. The synthesis of triazole moieties opens gates for the production of a series of derivatives containing various substituents to test their effect on the biological activities of 1,2,3-triazoles containing oxime [13].

### 1.1. Target Selection

**Figure 1:**
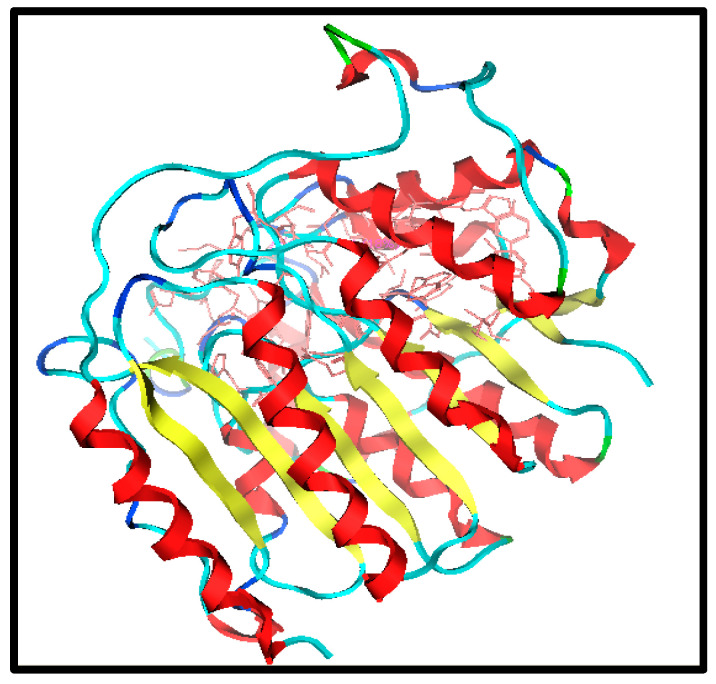
Structure of target (PDB ID 1T69).

The first step is to identify and select the most appropriate drug target to initiate the drug design. It is possible to identify the required protein target specifically linked to human diseases using bioinformatics tools. Insulin protein is considered a target protein for this study. Its structure is taken from the Protein Data Bank (PDB) under PDB ID 1T69, as shown in Figure 1.

### 1.2. Dataset Collection

The three standard drugs for diabetes were selected from PubChem. It is a public database that contains validated chemical structures and detailed information on drugs. The test set was selected from the literature, consisting of 55 (KS-1 to KS-55) compounds. The 2D structures of all 55 compounds were drawn in ChemDraw Ultra and saved as two file types: ChemDraw (*.mnx) and MDL Mofile (*.h.mol). The antidiabetic target protein (PDB ID 1T69) with the lowest resolution was selected and the 3D structures were obtained from the PDB (Protein Data Bank). The 2D and 3D structures of the antidiabetic standard drugs were retrieved from PubChem (Table 1). In addition, energy minimization for the target and dataset was performed on MOE using force field MMFF94x, and the dataset structures were saved in the .mdb file format.

### 1.3. Lead Identification and Analogue Design

The most important step was the hit optimization and lead identification. This was demonstrated based on three concepts. The predictive ability of the docking was assessed using the Root mean square deviation (RMSD) of the top-ranked solution. The most active compound was assessed in terms of the greatest number of interactions and binding behavior.

Following the lead selection which contained the core part of the triazole moiety, various functional groups were introduced and removed to create analogs.

## 2. Results and Discussion

### 2.1. Virtual Screening for Antidiabetic Compounds

**Figure 2:**
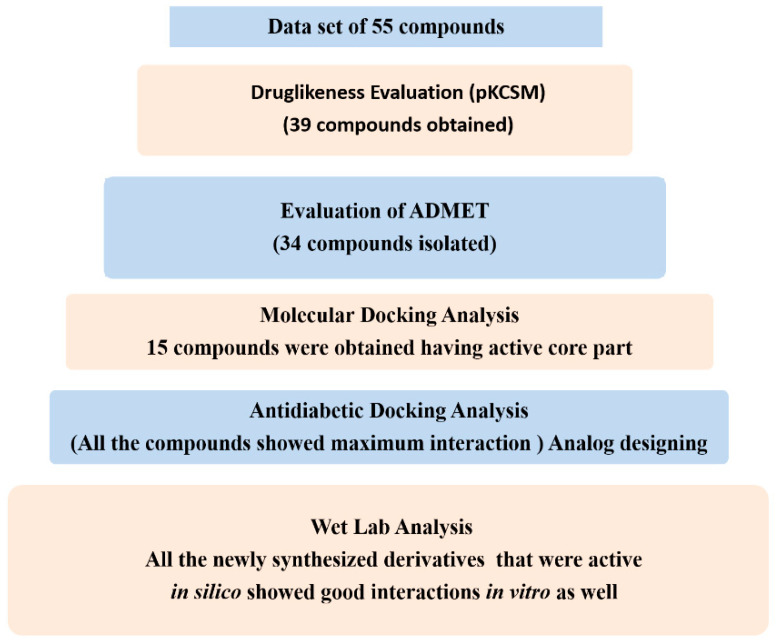
Summary of dry and wet lab analyses.

A molecular docking analysis of the selected 55 compounds, i.e., KS-1 to KS-55, was performed along with ones for the three standard drugs [14]. The target protein (PDB ID 1T69) was used as a molecular target. Based on the evaluation process, the docking modules perfectly docked 70–80% of the ligands [15,16,17,18]. The Molecular Operating Environment (MOE 2016) was employed for the molecular docking analysis of the compounds based on the 1,2,3-triazole moiety [19,20,21]. First of all, the docking log files were generated and then, one by one, the ligands present in the dataset were employed for docking in the target protein. Each compound was studied for docking and confirmed ten times. Afterwards, based on the lead compounds, the active compounds were synthesized and their docking behavior was studied. The generated confirmations of both the dataset and synthesized compounds were automatically categorized in ascending order based on the binding interactions and binding energies of the ligand with the target protein. The best confirmation was chosen on the basis of the docking analysis among all of the generated confirmations. The confirmation with the lowest binding energy was considered the best confirmation as shown by the flow chart in Figure 2.

### 2.2. Drug-Likeness and ADMET Properties

The molecular descriptors were calculated by using online software, i.e., SwissADME and pkCSM, as mentioned in the literature [22]. The training set of 55 compounds (KS-1 to KS-55) was selected from two different classes. All of the compounds were screened for their drug-like properties. Lipinski’s rule of five parameters was observed for each compound in the training set (Table 2). The results for the drug-likeness properties indicated the drug-like behavior of the candidates. Out of the 55 compounds of the training set, 39 drug-like candidates were selected. The compounds violating more than one rule of the five Lipinski parameters were eliminated from the dataset. The remaining 39 compounds were used for further pharmacokinetics studies. Compounds were further screened on the basis of their ADMET properties.

Following the drug-likeness study, the absorption, distribution, metabolism, excretion, toxicity, and ADMET properties were predicted for the 39 selected compounds. Based on intestinal absorbance, metabolism, and excretion, eight compounds were eliminated. The remaining 34 compounds were selected for further molecular docking studies. All of these 34 compounds were isolated on the basis of having good intestinal absorbance, metabolism and excretion. The values for intestinal absorption ranged from 72.063 to 99.985. The minimum and maximum values for excretion ranged from 0.13 to 0.721.

The numeric values of the compounds KS-09, KS-10, KS-13, KS-14, KS-44, KS-45, KS-48 and KS-49 were far from good in terms of intestinal absorption and excretion. Following the calculation of drug-likeness and the ADMET properties, 34 compounds were studied for the docking analysis. The compounds had no hepatotoxicity. On the basis of these properties, new derivatives were designed and synthesized. The drug-likeness and ADMET properties of the synthesized compounds are given in the tables below. The results indicated that the synthesized compounds behaved as drug-like candidates with no hepatotoxicity and very little oral toxicity (Table 3).

### 2.3. Molecular Docking Analysis

The main outcome of the molecular docking was to identify the best binding interaction between the target protein and the synthesized ligands. The structure of the targeted antidiabetic protein was obtained from the RCSB Protein Data Bank (PDB), PBB ID 1T69. In order to control the performance of our docking approach, in the case of 1T69, the crystal structure of the 1T69 protein was selected along with its active site, which was found to be located at the bottom of a deep and narrow gorge [23]. The key contributing amino acids along with their interactions with the designed analogues are described in Table 4 below.

**Figure 3:**
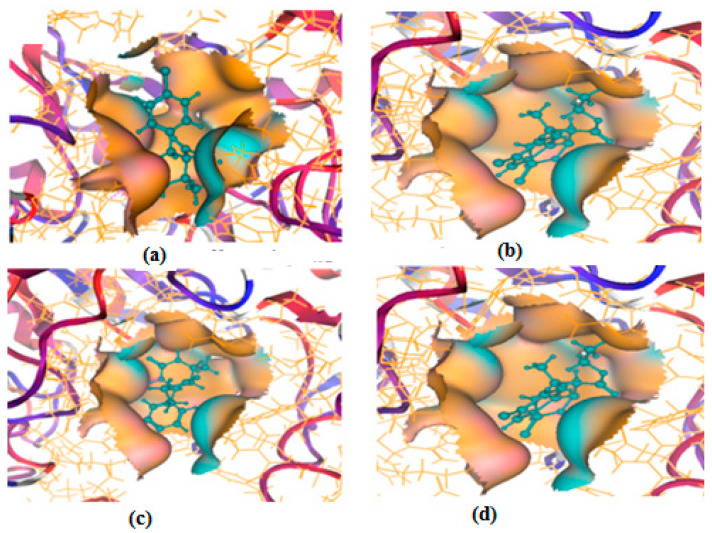
3D docking structure of lead compounds (**a**) K-1, (**b**) S-2, (**c**) K-2, (**d**) H-4 in the active site of 1T69.

All of the four analogues designed were found to be active after the docking analysis on the basis of having the best binding interactions as shown in Figure 3. Compound K-1 had the maximum number of hydrophilic interactions via the O-atom and N-atom with the core amino acids. Compound K-2 had both arene–pi and hydrophilic interactions with the surrounding amino acids. Similarly, compound S-2 had hydrophilic interactions only, and H-4 had both arene–pi and hydrophilic interactions within the minimum possible distance, as described in Table 4. The compound K-1 had the maximum binding interactions with the minimum binding energy. The heteroatoms, such as N and O, present in the ligand K-1 and the key contributing amino acids of compound K-1 had good antidiabetic behavior. 

**Figure 4:**
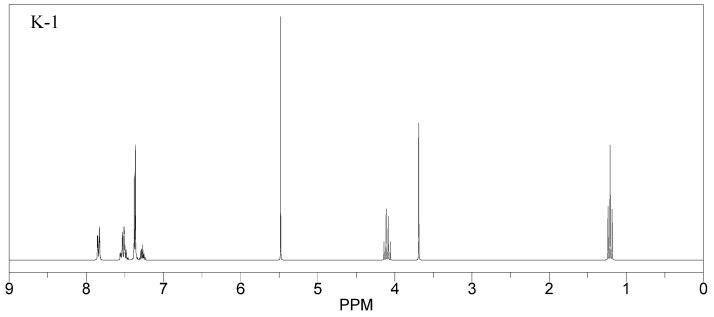

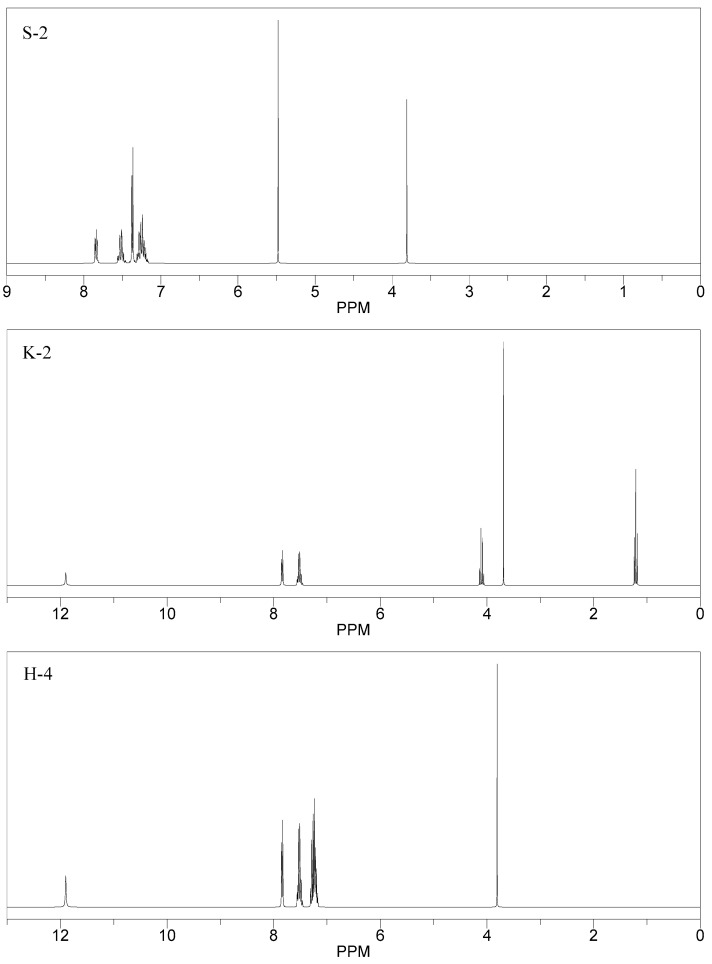
^1^H-NMR spectra of synthesis compounds.

All the compounds were synthesized according to the procedure mentioned in the literature and were characterized by FTIR spectroscopy, mass spectroscopy and H-NMR spectroscopy (Figure 4). The FTIR spectral data of triazoles (K-1) exhibited characteristic C=O absorption at 1600 cm^−1^. The other peaks were observed as aromatic C=C at 1569 cm^−1^, C-N at 1160 cm^−1^ and C-H stretching at 757 cm^−1^. The FTIR spectral data of K-2 exhibited characteristic aromatic C-H absorption at 735 cm^−1^. The other peaks were observed as aromatic C=C at 1569 cm^−1^, C-N at 1162 cm^−1^ and sp^2^ C-H stretching at 3170 cm^−1^. The FTIR spectral data of S-2 exhibited characteristic aromatic C=O absorption at 1634 cm^−1^. The other peaks were observed as aromatic N=N at 1220 cm^−1^, aromatic C-H at 751 cm^−1^ and aliphatic C-H stretching at 2341 cm^−1^. The FTIR spectral data of H-4 exhibited characteristic aromatic C-H absorption at 737 cm^−1^. The other peaks were observed as aromatic C=C at 1568 cm^−1^ and C-N at 1157 cm^−1^. The m/z peaks of the compounds K-1, K-2, S-2 and H-4 were obtained as 322, 232, 250 and 322, respectively. The peaks were in the form of (M + H). All the synthesized compounds were obtained in good yields in the range of 56–79% and they were characterized by their physical constants and spectroscopic data. The melting points of all the compounds were recorded and found to be in the range of 140–290 °C.

### 2.4. In Vitro Antidiabetic Assay 

All synthesis and characterize compounds were further evaluated and alpha amylase and alpha glucosidase inhibition assays (as shown in Table 5 and Table 6), the results corresponded closely to the the dry lab results, as the K-1 had the maximum percentage of inhibition in both of the assays. The compounds K-1 S-2 K-2 and H-4 had more than 80% inhibition of alpha amylase at the concentration of 800 µg/mL, showed promising antidiabetic activity and can be used as structural models for developing better antidiabetic agents. Out of these four compounds, the compound K-1 had the maximum antidiabetic activity.

The current study involved both dry and wet lab analyses of multiple rounds of experiments, as discussed in the above sections. The in vitro studies showed that all the synthesized compounds showed antidiabetic potential. The antidiabetic activity of these four compounds was already predicted in molecular docking studies. Hence, all the tests performed for these compounds showed they had good binding energies and all the necessary chemical features required for binding in the active site. It was observed that all the active compounds were deeply embedded in the active site of 1T69 and all these compounds were stabilized by the presence of hydrophobic and hydrophilic interactions. The antidiabetic activity of the synthesized compounds was confirmed via age (%) inhibition assays as well.

## 3. Materials and Methods

### 3.1. Materials

Benzyl azide, sodium azide, and benzyl chloride were purchased from J&K Scientific from China and were used without further purification. Copper phenylacetylide was prepared according to the procedures reported in the literature [24]. Solvents such as tetrahydrofuran (THF), chloroform, petroleum ether and ethylacetate of analytical reagent (AR) grades were purchased from Sigma Aldrich, Saint Louis, MO, USA and used without purification.

### 3.2. Experimental Equipment

The synthesized compounds were characterized by FT-IR, H-NMR and mass spectrometry. ^1^H NMR spectra were recorded on a Bruker, San Jose, CA, USA spectrometer at 300 MHz, respectively, in DMSO solution. In this study, solvent was used as an internal reference. Chemical shifts were given at δ scale (ppm), and abbreviations s, d, t, q and m were used for singlet, doublet, quartet and multiple, respectively. Coupling constants were presented in Hz. Mass spectra of the new synthesized compounds were recorded on Agilent Technologies 6890 N, Santa Clara, CA, USA inert mass selective detector. The melting points of the chemical compounds were determined in open capillaries using Gallenkamp, Cambridge, US melting point (MP-D). FT-IR spectra of all the compounds were recorded in the 4000–380 cm^–1^ range with samples in KBr discs via a PerkinElmer, Waltham, MA, USA apparatus.

### 3.3. Docking Procedure

Molecular docking analysis was carried out using MOE-Dock (Chemical Computing Group Inc.) on a machine with a Pentium 1.6 GHz workstation with 512 MB of memory using the Windows operating system. Docking studies were executed in order to study the drug correlation and ADMET parameters for antidiabetic activity. All of the steps in docking studies were performed by the procedure in the literature [23]. These steps included target preparation, docking and scoring. The crystal structure of the protein against type-II diabetes was downloaded from Protein Data Bank (PDB) and the pdb ID was 1T69. The protein structure was imported into MOE. The target structure was protonated in 3D and its energy was minimized, eliminating the water molecules and adding all hydrogen atoms with the standard structural geometry. The site finder tool was used to search the systematic conformation of the resulting model at default parameters with root mean square (RMS) gradient of 0.0001 kcal/mol. The active site of the protein was searched by the site finder. In order to identify the active sites, dummy atoms were created from the resulting alpha spheres. The energy was minimized while the backbone and residues were kept fixed. RMSD values were used to compare the ligand between the predicted and its corresponding crystal structure. The resulting docked poses were clustered together with RMSD less than 1.3 Å.

Docking analysis was conducted, and 10 conformations for each ligand were generated by using MDB file. The energy minimization for the target and dataset was performed on MOE using force field MMFF94x, and the dataset structures were saved in the .mdb file format. Molecular docking was performed between the dataset and the target. The best (in terms of energy minimization) conformation of the ligands was selected out of ten conformations on the basis of their binding interactions and lowest binding energy. As a result, among these confirmations, the lowest energy confirmation, besides those of all the ligands, was selected and appended at the end of the original protein file. This process produced docked files based on the molecular operating environment for the specific set.

### 3.4. Synthesis of the Hits Identified

#### General Procedure for the Synthesis

**Figure 5:**
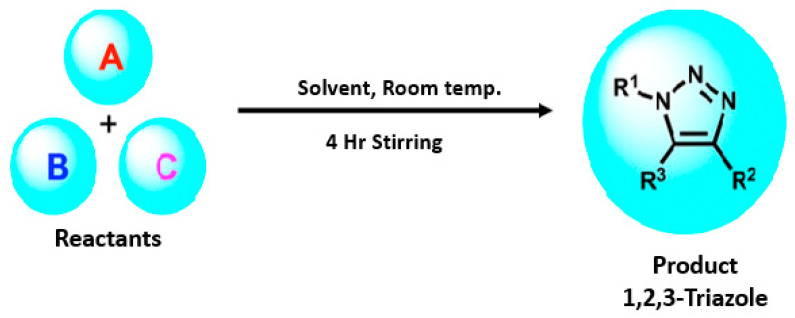
General scheme for the synthesis of triazole.

All the triazole derivatives were synthesized by the procedure mentioned in the literature with few modifications [24]. In this case, tetrahydrofuran (THF) served as solvent, and all the reactants, A (0.08 g), B (0.09 g) and C (0.07 g), were added into one pot and allowed to react upon stirring for 4 h at room temperature (Table 7, Figure 5). The reaction was followed by flash column chromatography elution with 10% ethyl acetate in petroleum ether. After stirring it for four hours, the product was obtained. In some cases, the product was recrystallized using the solvent. Triazoles (K-1 to H-4) were characterized through their physical constants, and their melting points were found to be in agreement with those of the literature [12].

### 3.5. In Vitro Analysis

#### 3.5.1. α-Amylase Inhibition Assay

α-Amylase is considered to be an endoamylase, and it can be purified from the salivary gland and pancreas by various purification methods. By catalyzing the starch’s breakdown into maltose, it is further hydrolyzed, producing glucose that can be absorbed in the blood. The glucose level is precisely regulated in the blood, as slight increase in the blood glucose level could in turn cause hyperglycemia. Inhibitors of alpha-amylase are an important therapeutic approach for lowering elevated blood glucose levels in post-prandial hyperglycemia. This research paper suggests that the presence of specific group containing this compound may have potentially important role in managing hyperglycemia via inhibiting alpha amylase. In this assay, acarbose is used as a standard antidiabetic drug and the activity of the synthesized compounds is measured as compared to acarbose [25,26].

**Figure 6:**
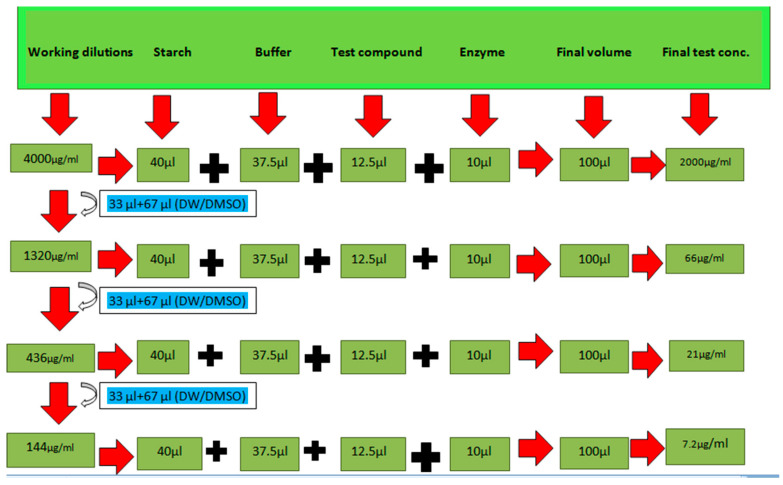
Pictorial description of working dilutions taken in alpha amylase inhibition assay [4,5].

The assay was performed in 96-well microliter plates [25]. To prepare the reaction mixture, 37.5 µL of phosphate buffer (pH 6.8), 10 µL of the enzyme, 12.5 µL of the sample (distilled water/DMSO)/acarbose and 40 µL of starch were added to wells. It was then incubated at 50 °C for 30 min. The reaction was terminated by the addition of 20 µL of 1 M HCl. A total of 100 µL of iodine was added to all the wells and the microplate reader measured absorbance at 540 nm [26] as shown by pictorial representation in Figure 6. The inhibition percentage was calculated by using the following formula:

$$\mathrm{Percentage}\;\mathrm{Inhibition}=\left[1-\frac{Absorbanceofuntreatedcontrol}{Absorbanceofsample}\right]\times 100$$

#### 3.5.2. Alpha-Glucosidase Inhibition Assay

Alpha-glucosidase inhibitory activity was determined according to the previously reported method with minor modifications [26]. The reaction mixture was prepared in a 96-well plate by adding the following chemicals in sequential order: 25 μL p-NPG (20 mM), 69 μL phosphate buffer (50 mM, pH6.8) and 1 μL α-glucosidase enzyme (3 U/mL). They were mixed in a 96-well microtiter plate. Five μL of the compound with final concentrations of 800, 400, 200, 100, 50, 25, 12.5 and 6.25 μg/mL was added into respective wells. Acarbose and DMSO were used as positive and negative controls, respectively. The mixture prepared in 96-well plate was incubated at 37 °C for 30 min, followed by the addition of 100 μL of NaHCO_3_ (0.5 mM) to stop the reaction. The absorbance was measured at 405 nm using a microplate reader (BioTek Elx-800, Winooski, VT, USA).

Inhibitory activity (%) = (AC – AS)/AC × 100

where AS is the absorbance in the presence of the test substance, and AC is the absorbance of control.

## 4. Conclusions

The aim of the current study was to systematically and methodically find out the lead compounds that can act as strong antidiabetic agents. Different types of binding interactions were observed between the designed and synthesized compounds with the target protein 1T69 during in silico analysis and have shown good responses for in vitro inhibition assays as well. All the compounds were found to be active, as predicted by dry lab approaches which lent a hand in the suppression of DM type-II. Out of these compounds, the compound K-1 has the maximum antidiabetic activity. These compounds may function as a starting point for our understanding of promising antidiabetic agents.

## Figures and Tables

**Table 1:** Chemical structures and IC_50_ values of the training set.

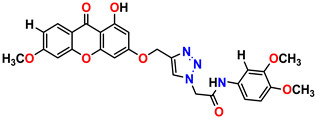 Compound **1** **KS-1**, 15.90 ± 0.91 µM	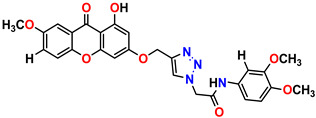 Compound **2** **KS-2** > 100 µM
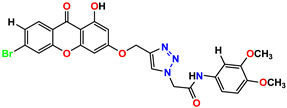 Compound **3** **KS-03**, 11.82 ± 0.82	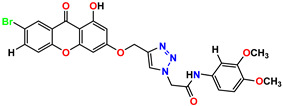 Compound **4** **KS-04**, 29.84 ± 3.47
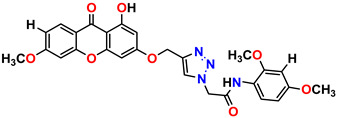 Compound **5** **KS-05**, 2.06 ± 0.16	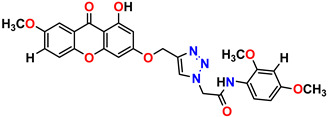 Compound **6** **KS-06**, 8.31 ± 0.88
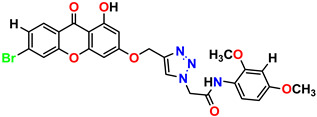 Compound **7** **KS-07**, 2.78 ± 0.22	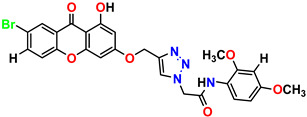 Compound **8** **KS-08**, 3.07 ± 0.56
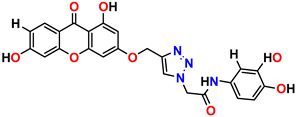 Compound **9** **KS-09**, 6.13 ± 0.09	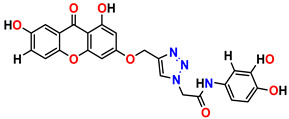 Compound **10** **KS-10**, 7.06 ± 0.89
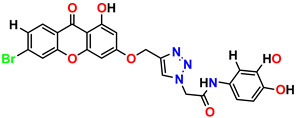 Compound **11** **KS-11**, 5.23 ± 0.53	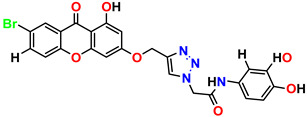 Compound **12** **KS-12**, 3.17 ± 0.61
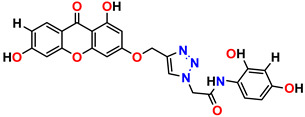 Compound **13** **KS-13**, 17.61 ± 1.68	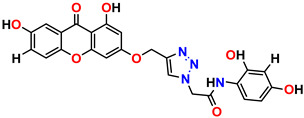 Compound **14** **KS-14**, 15.62 ± 1.13
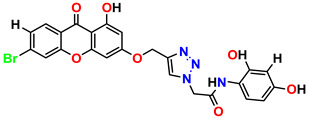 Compound **15** **KS-15**, 5.87 ± 0.76	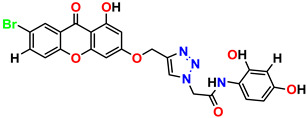 Compound **16** **KS-16**, 5.88 ± 0.32
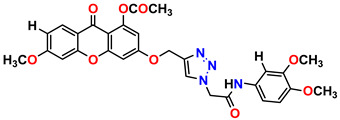 Compound **17** **KS-17**, 98.63 ± 4.12	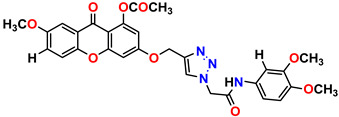 Compound **18** **KS-18**, >100
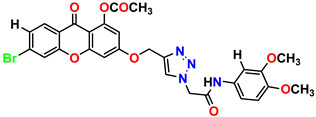 Compound **19** **KS-19**, 26.11 ± 2.95	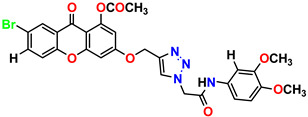 Compound **20** **KS-20**, 32.33 ± 0.82
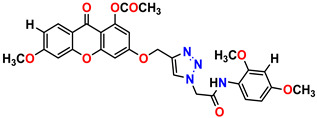 Compound **21** **KS-21**, 33.16 ± 2.51	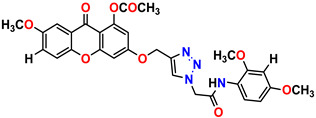 Compound **22** **KS-22**, 42.60 ± 0.09
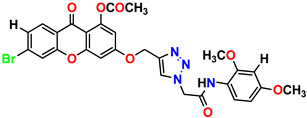 Compound **23** **KS-23**, 12.78 ± 0.01	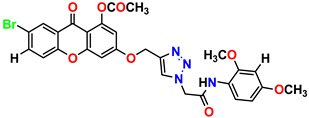 Compound **24** **KS-24**, 12.13 ± 1.84
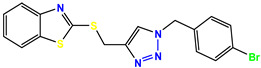 Compound **25** **KS-25**, 28.7	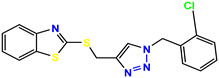 Compound **26** **KS-26**, >100
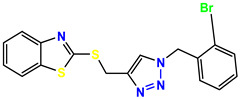 Compound **27**, **KS-27**, 37.4	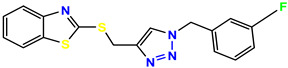 Compound **28**, **KS-28**, 61.1
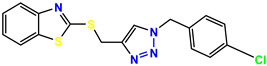 Compound **29** **KS-29**, 27.4	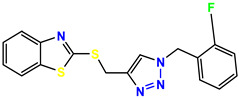 Compound **30** **KS-30**, >100
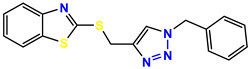 Compound **31** **KS-31**, >100	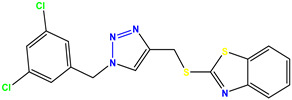 Compound **32** **KS-32**, 41.0
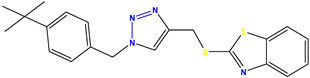 Compound **33** **KS-33**, 29.4	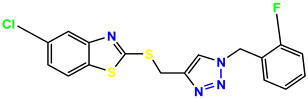 Compound **34** **KS-34**, 45.9
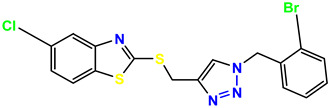 Compound **35** **KS-35**, 48.4	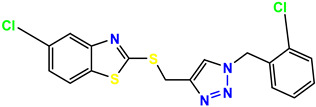 Compound **36** **KS-36**, 33.6
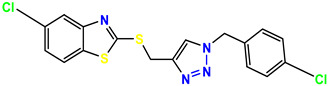 Compound **37** **KS-37**, 28.2	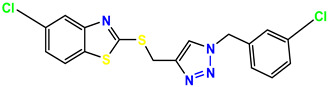 Compound **38**, **KS-38**
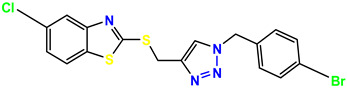 Compound **39** **KS-39**	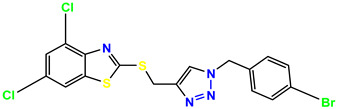 Compound **40** **KS-40**
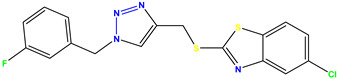 Compound **41** **KS-41**	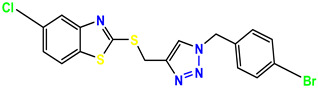 Compound **42** **KS-42**
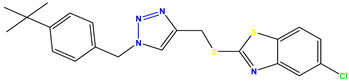 Compound **43** **KS-43**	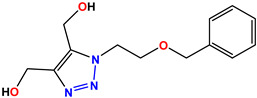 Compound **44** **KS-44**
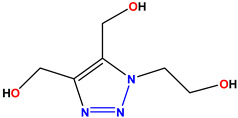 Compound **45** **KS-45**	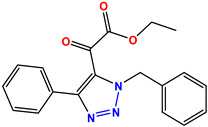 Compound **46** **KS-46**
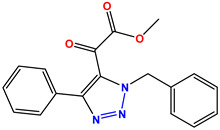 Compound **47** **KS-47**	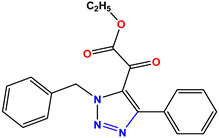 Compound **48** **KS-48**
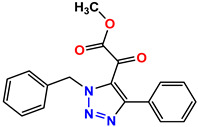 Compound **49** **KS-49**	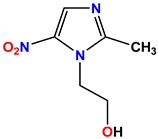 Compound **50** **KS-50**
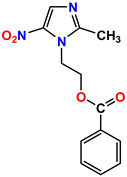 Compound **51** **KS-51**	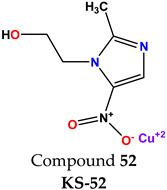 Compound **52** **KS-52**
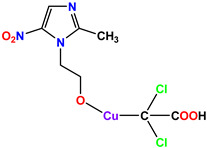 Compound **53** **KS-53**	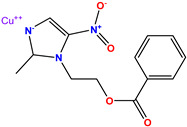 Compound **54** **KS-54**
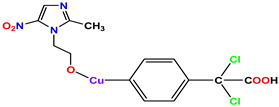 Compound **55** **KS-55**	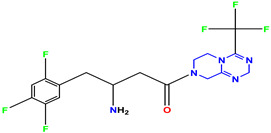 Standard **1** Sitagliptin
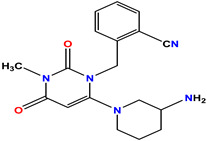 Standard **2** Alogliptin	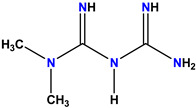 Standard **3** Metformin

**Table 2:** Drug-likeness of synthesized compounds.

Sr. No	Molecular Wt. g/mol	Log P	Rotatable Bonds	Acceptors	Donors	Surface Area (g/cm^2^)
01	321.4	3.1	6	5	01	140.4
02	231.3	2.5	4	5	0	99.2
03	249.3	2.9	4	3	0	112.1
04	235.3	3.7	3	3	0	105.9

**Table 3:** Pharmacokinetics of the synthesized compounds.

Sr. No	Absorption mg/L	Distribution mg/L	Metabolism mg/L	Excretion mg/L	Toxicity Oral/Hepatotoxicity
01	98.726	0.128	Yes	0.394	No/No
02	94.696	0.271	Yes	0.327	0.23/No
03	97.12	0.088	Yes	0.272	No/No
04	94.539	0.191	Yes	0.28	0.8/No

**Table 4:** Binding interactions and binding energy of synthesized compounds.

Sr. No	Compounds	Hydrogen Bonding		Arene–pi Interactions	Binding Energy (S)
01	**K-1**	2.74, 2.86 1.13, 2.64 2.59	Tyr18, Ser39, Tyr24, Arg37, Asn136	**--**	−0.6467
02	**S-2**	2.48, 1.8, 2.52	Lys36, Ser39	Yes	−4.0720
03	**K-2**	2.47, 2.02	Lys33, Trp141	**--**	−3.0564
04	**H-4**	2.32, 1.15	Gyl151, Ser150	yes	−2.5427

**Table 5:** Results of alpha amylase inhibition assay.

Dose µg/mL	%Age Inhibition at Different Concentration							
	800	400	200	100	50	25	12.5	6.25
**K-1**	87.012	81.11	74.89	64.24	55.81	42.21	37.23	23.11
**S-2**	84.22	78.56	71.59	59.1	52.22	44.81	32.22	21.47
**K-2**	81.89	73.45	58.23	48.56	39.76	29.34	20.42	12.78
**H-4**	83.12	76.11	64.72	53.12	45.12	39.12	25.1	16.89

**Table 6:** Results of alpha glucosidase inhibition assay.

	%Age Inhibition at Different Concentrations							
Dose µg/mL	800	400	200	100	50	25	12.5	6.25
**K-1**	99.17	97.01	95.55	91.11	88.96	83.11	72.37	68.04
**S-2**	±96.22	±95.01	93.81	90.24	86.02	72.41	69.23	65.10
**K-2**	87.19	83.12	71.03	65.10	57.8	51.69	44.32	31.09
**H-4**	89.12	81.01	75.00	67.11	55.21	49.11	40.81	41.09

**Table 7:** List of reactants and products with percentage yield.

Samples		Reactants				Product		Yield
	A	B	C	R_1_	R_2_	R_3_		
K-1	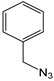	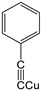	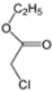	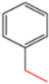	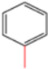	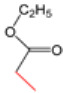	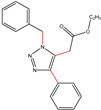 ethyl 2-(1-benzyl-4-phenyl-1 *H*-1,2,3-triazol-5-yl)acetate	79%
S-2	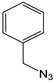	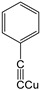	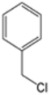	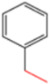	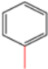	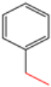	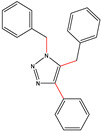 1,5-dibenzyl-4-phenyl-1*H*-56%1,2,3-triazole	71%
K-2	NaN_3_	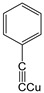	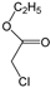	H	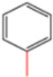	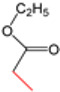	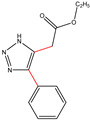 ethyl 2-(4-phenyl-1*H*-1,2,3-triazol-5-yl)acetate	56%
H-4	NaN_3_	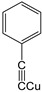	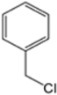	H	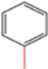	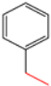	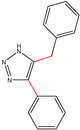 5-benzyl-4-phenyl-1*H*-1,2,3-triazole	67%

## Data Availability

Not applicable.

## Sample Availability

Samples of the compounds are available from the authors.
